# Physicochemical, sensory and microbiological characteristics of coriander seed powder yogurt

**DOI:** 10.1186/s13568-023-01572-5

**Published:** 2023-06-28

**Authors:** Naiema Vakili Saatloo, Tooraj Mehdizadeh, Javad Aliakbarlu, Rahele Tahmasebi

**Affiliations:** 1 Department of Food Hygiene and Quality Control, Faculty of Veterinary Medicine, Urmia University, Urmia, Iran; 2 Research and Department of Chromatography, Iranian Academic Center for Education, Culture and Research (ACECR), Urmia, Iran

**Keywords:** Synbiotic yogurt, Probiotic, Prebiotic, Coriander

## Abstract

Yogurt is a fermented food obtained by the bacterial fermentation of milk. In the present work, the effect of different concentrations (1, 3 and 5% w/w) of coriander (*Coriandrum sativum*) seed powder on physicochemical, sensory characteristics and viability of *Bifidobacterium bifidum* and *Lactobacillus acidophilus* of probiotic yogurt were investigated at 4 °C for 21 days. Laboratory-made yogurts were obtained by inoculating milk with yogurt bacteria (mixed culture of *Streptococcus thermophilus* and *Lactobacillus delbrueckii ssp. bulgaricus*) and two probiotic cultures (*Lactobacillus acidophilus* and *Bifidobacterium bifidum)*. According to the results, the viability of *B. bifidum* and *L. acidophilus* increased in synbiotic stirred yogurts with 5% CSP (coriander seed powder) up to 9.15 ± 0.09 log CFU/g at 11 days of the storage period, whereas probiotic bacteria count decreased to 9.02 ± 0.01 by the end. Therefore, our results confirmed that the addition of probiotics and CSP powder improved the physicochemical and sensory characteristics of stirred yogurt and exerted a beneficial effect on probiotic bacteria.

## Introduction

Probiotics are live microorganisms that are intended to have purposive benefits in admitted amounts when applied to the body (Gopal 2020). Different aspects of food safety, functional and technological impacts occur by the selection of probiotics in dairy and nondairy fermented products (Khaled 2021). Due to the health benefits of probiotics in human and animal bodies, expansion of agricultural demands for probiotics (animal, fish, and plant production) have occurred. Common, beneficial bacteria which have a long-standing association with health include lactic acid producing genera such as the Bifidobacteria or Lactobacilli. Lactic acid bacteria and bifidobacteria are presented as starter cultures and commercial probiotics, used in fermented products such as yogurt and milk (Kycia et al. 2020). To the success of the healthy advantages, keeping high levels of probiotic bacteria (6–7 log CFU/g) at the consumption step is essential (Mehdizadeh et al. 2019). Viability of the cells is necessary when the product is consumed. Yogurt has been considered a proper matrix for the availability of probiotic microorganisms. As the growing fame of yogurt, finding valuable ingredients such as probiotics and prebiotics to procreate functional yogurt is pursued (Mehdizadeh et al. 2021).

According to the increasing applications of pro- and prebiotic products, considering human or animal health is important (Zoumpopoulou et al. 2018). Prebiotics are nondigestible carbohydrates that selectively stimulate the growth or activity of desirable microorganisms (Macfarlane and Cummings 1999). When they reach the large intestine, rolling as nutritional substrates for beneficial intestinal bacteria. The demand for prebiotic products as functional food components has been growing and worldwide prebiotic trading is expected to reach 7.2 billion USD by 2024 (Cardoso et al. 2021).

Besides probiotics and prebiotics being widely used, synbiotics are studied for their beneficial effects on host health. Synbiotics combine the pro- and prebiotic components, somehow that they act synergistically. In such preparations, prebiotics not only stimulate the growth of the beneficial microorganisms residing in the gastrointestinal tract (GIT), but they also enhance the survival of the included probiotics (Szlufman and Shemesh 2021). Therefore, an appropriate mixture of both components in a single product should affirm a preferable effect, compared to the activity of the probiotic or prebiotic separately (Markowiak and Śliżewska 2017).

Coriander (*Coriandrum sativum* L.), known as cilantro, is an annual herb belonging to the Apiaceae family. It has also been used as an aromatic plant for centuries (Rabiei et al. 2020). This plant is cultivated where the climate favors its growth, particularly in Iran, Indonesia, Russian, Afghanistan, China, India, Tanzania, Turkey, and Bulgaria (Ashraf et al. 2020).

Coriander seeds and the fruit of coriander, are soft, weightless and valuable parts of this plant due to the presence of characteristic aroma compounds (about 1.8% essential oil). They are mainly processed into powder by crushing, and this powder, with its aroma, is used as a food ingredient. The seeds are also used to extract essential oils (Hosseinzadeh et al. 2014). In traditional medicine, coriander was used for various ailments, including gastrointestinal issues, respiratory problems, and pain relief. Ongoing research is exploring its potential uses as an aphrodisiac and appetite stimulant, hypoglycemic and hypolipidemic effects, diabetes-modulating, and neurological benefits (Anwar et al. 2023). Furthermore, it has been used as an antifungal, antioxidant and hypolipidemic component (Hosseinzadeh et al. 2014). The antioxidant properties of most plants are due to the presence of polyphenols (Sadighara et al. 2016). Polyphenol fraction of *Coriandrum sativum* seeds is due to nine molecules (Vanillic acid: C_8_H_8_O_4_, chlorogenic acid: C_16_H_18_O_9_, catechin: C_15_H_14_O_6_, epicatechin: C_15_H_14_O_6_, oleuropein: C_25_H_32_O_13_, epicatechin gallate: C_22_H_18_O_10_, rutin: C_27_H_30_O_16_, gallocatechin: C_15_H_14_O_7_, epigallocatechin: C_22_H_18_O_11_ (Mechchate et al. 2021). Its phytochemical content, includes phenolic compounds, flavonoids, ascorbic acid and carotenoids (Ahmed et al. 2018). In vitro studies suggested that polyphenols have to limit or inhibit the growth of detrimental species, such as Clostridiales and Enterobacteriales and favor the growth of beneficial bacteria, such as Lactobacillus spp. and Bifidobacterium spp (Nazzaro et al. 2020). Polyphenols can play an important role as prebiotics, in addition to their well-established properties, while a synergistic effect between prebiotic polyphenols and probiotic bacteria may occur (Gibson et al. 2017).

Consumers want safe and healthy foods. Functional foods can contain instance probiotics (living bacteria), prebiotics and antioxidants (Hoseinifar et al. 2021). The biological activities of coriander are the main reason to increase in its potential uses as a functional food for the health-giving additives industry (Sahib et al. 2013). Therefore, this study aimed to examine the effects of using different levels of coriander seed powder on the viability and survival of *Lactobacillus acidophilus* and *Bifidobacterium bifidum* in stirred coriander yogurt throughout storage.

## Materials and methods

### Bacterial culture

The starter culture (containing *L. delbrueckii ssp. bulgaricus* and *S. thermophilus*) was obtained from Chr. Hansen (Copenhagen, Denmark). The lyophilized culture of *L. acidophilus* PTCC1608 and *Bifidobacterium bifidum* PTCC1644 was bought from Persian Type Culture Collection (Tehran, Iran). To produce an active bacterial culture, the lyophilized powders of *L. acidophilus* and *B. bifidum* were transferred to the tubes containing 5ml MRS broth and then incubated at 37 °C under anaerobic conditions for 24 h, then cultured on MRS agar plates for counting Commercial MRS agar (Merck, Darmstadt, Germany), Commercial MRS broth (Merck, Darmstadt, Germany) and MRS-bile agar medium (MRS agar: Merck, Darmstadt, Germany and Bile: Sigma, Reyde, USA) were rehydrated in distilled water according to manufacturer’s instructions. MRS agar was used as a fundamental medium to formulate selective media compounds. Bacteriological peptone diluent (0.1%) was prepared by dissolving 1 g of peptone (Oxoid) in 1000 mL of distilled water. The pH value was adjusted to 7.0 0.2 at 25 °C, followed by autoclaving 9 mL aliquots at 121 °C for 15 min.

### Yogurt production

To prepare standard yogurt (two types of yogurt including probiotic and coriander seed powder), fresh milk was obtained from the local dairy farm. The fermentation process was carried out using commercial starter culture (*Streptococcus thermophilus* and *Lactobacillus delbrueckii subsp. Bulgaricus*) following the manufacturer’s recommendation. The milk samples were heated to 85 °C for 20 min for pasteurization. After cooling to 42 °C, 2% w/w of the starter culture (containing *Lactobacillus delbrueckii ssp. bulgaricus* and *Streptococcus thermophilus*) and 1% w/w of the *Lactobacillus acidophilus* and *Bifidobacterium bifidum* suspension, as the probiotic strain, were added. The samples were placed in an incubator at 42 °C until the pH decreased to 4.6 (3–4 h). After this step, yoghurt samples were kept at 4 °C for 1 day. For synbiotic yogurt production, coriander seed powder (1,3 and 5% w/w) was added to set yogurt. The *L. acidophilus* and *Bifidobacterium bifidum* count, the physicochemical and sensory attributes of the yogurt samples were determined after 1, 11 and 21 days of refrigerated storage.

### The analysis of chemical components of coriander seed powder

Dried coriander seeds were homogenized to a fine powder. The seeds were washed well with water, air-dried at room temperature, and then ground in an electric grinder to have a coarse powder. The proximate analysis of coriander seed powder was defined (AOAC. Official Methods of Analysis of the Association of Official’s Analytical Chemists. 17th Edn. Association of Official Analytical Chemists).

### UV-C treatment on coriander seed powder

Dried coriander seeds (*Coriandrum sativum*) were obtained from a local herb shop and grounded to powder form. UV-C treatments were performed in a metal box (65 × 90 × 45 cm^3^) with slight modifications. Samples of coriander were packed in bags and exposed to the UV-C lamp (TUV-75 w G75 T8 220 V, Philips, Holland with peak emission at 254 nm). The UV-C dose inside the package during treatments 10.0 kJ m ^− 2^. The sample packages were located 15 cm from the UV-C source (Hassan et al. 2020).

### Enumeration of probiotic bacteria

Serial decimal dilutions of *L. acidophilus* and *B. bifidum* were obtained and plated on MRS Agar (McCoy and Gilliland 2007; Miranda et al. 2011).

### Physical and chemical characteristics

The measurement of pH was done by using a digital pH meter(Hanna, Fisher Scientific Company, Pittsburgh, PA) and the titratable acidity values of each yogurt sample were determined after mixing the yogurt samples with 10 mL of hot distilled water (90 °C) and titrating with 0.1 N NaOH containing 0.5% phenolphthalein as an indicator to an endpoint of the faint pink color (Noh et al. 2013). All samples were measured in triplicate.

To determine sample syneresis and water holding capacity using the method described by (Molaee Parvarei et al. 2021). Briefly, the released whey from yogurt samples, centrifugation was carried out (10 g sample 258× g – 10 min − 4 °C. The WHC of the yogurt samples was determined via centrifugation (10 g sample at 1613× g for 30 min, at 10 °C) according to Mortazavian et al.2020 (Molaee Parvarei et al. 2021). The supernatant solution was separated, and the resulting precipitate was weighted.

### Sensory evaluation

Five panelists from Urmia University assessed the yoghurt samples. Five-point hedonic scale test (including 0 = dislike very much; 1 = “dislike”, 2 = “neither like nor dislike”, 3 = like and 4 = like very much) was used for evaluating sample acceptability. The effect of coriander seed powder on the sensory properties of stirred, probiotic and synbiotic yoghurt samples was determined by five experienced panelists (3 females, 2 males; aged 25–45 years) and the rating information about the acceptance of the samples is obtained by using a numerical interval between 0 and 50 (mouth feels 0–14, non-mouth feel properties 0–8, flavor 0–24, appearance 0–4 and overall acceptability 0–50).

### Statistical analysis

Statistical analysis was performed using analysis of variance (ANOVA) according to a repeated measure experimental design with the aid of IBM SPSS Version 24. Results were compared using Tukey’s post-hoc test and considered significantly different at *p* < 0.05.

## Results

### The chemical composition of coriander seed

**Fig. 1:**
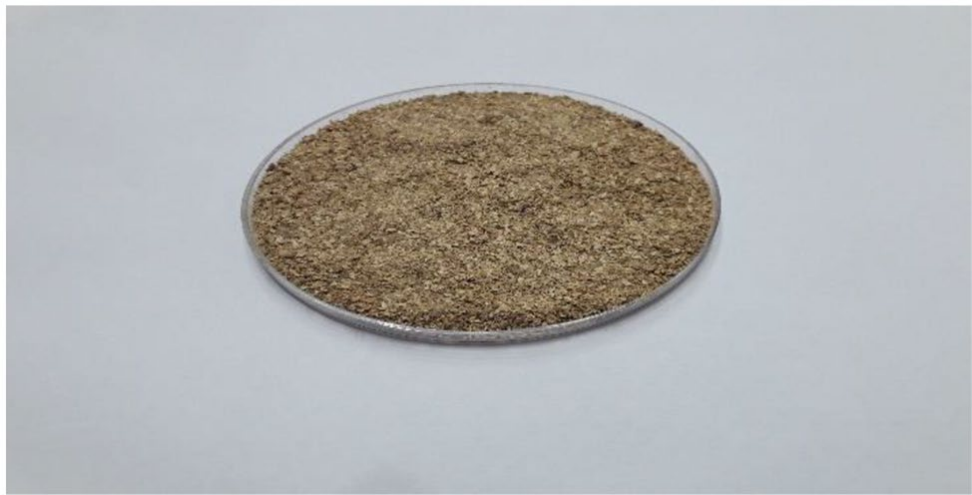
Coriander seed powder

The analysis of coriander powder (Fig. 1.) was determined, that dry matter 85%, Crude protein 14.12%, crude fiber 34.56, Ether extract 24%, Ash 11% (Tobaruela et al. 2018).

### Preparation of probiotic bacteria

**Fig. 2:**
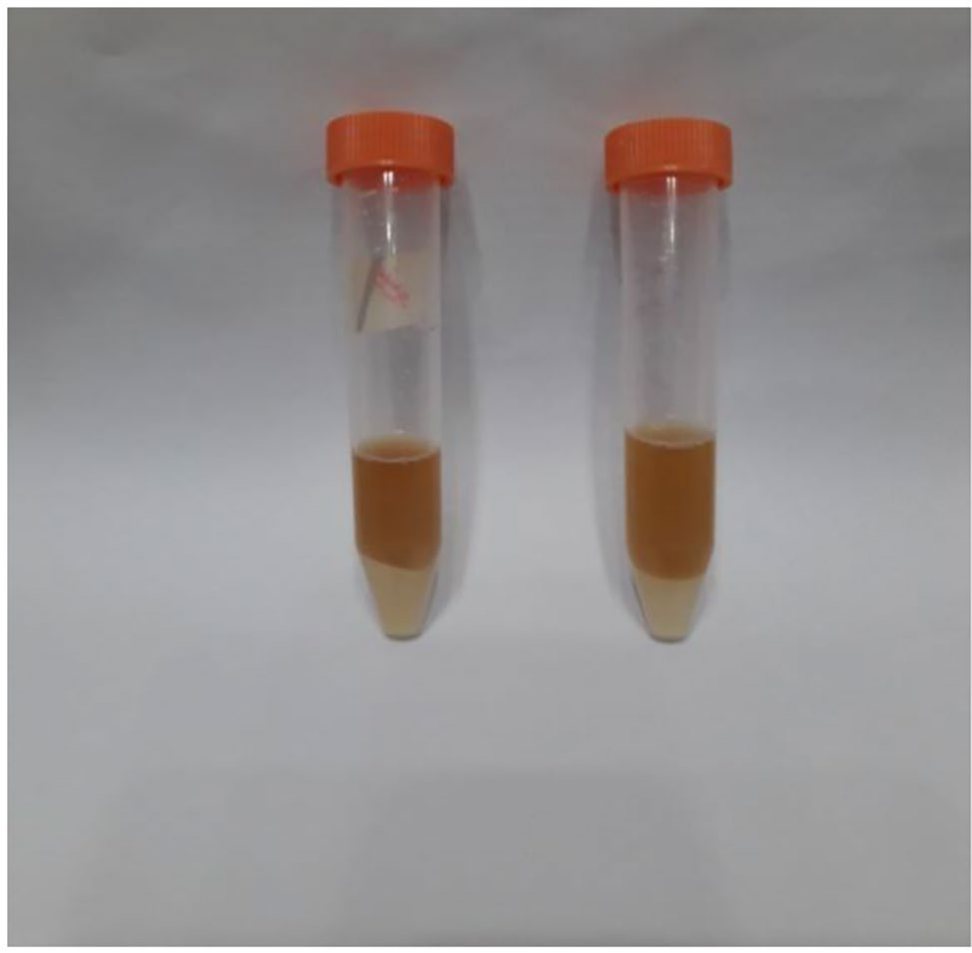
Sediment of *Bifidobacterium bifidum* and *Lactobacillus acidophilus* in MRS broth

Live bacteria were spun down by centrifuging at 12,000 ×g for 10 minutes. Supernatants were separated from spun-down bacteria, filtered, and diluted. Strains of probiotic bacteria were reactivated in MRS broth (at 37°C for 12 h with shaking), then live bacteria were spun down by centrifuging at 12,000 ×g for 10 minutes. Aliquots of probiotic bacteria in tubes with MRS broth media were incubated anaerobically at a temperature of 37°C for 24 h, then streaked on MRS agar and incubated at 37°C for 48 h. After incubation, the growth of microorganisms was shown as visible growth (Fig. 2.). The bacterial cell density of *Lactobacillus acidophilus* and *Bifidobacterium bifidum* were adjusted to 2.5×10^6^ cells/ml (OD_600nm_ = 0.255) and 8.6 × 10^6^ cells/ml (OD_600nm_ = 0.125) respectively) ascertained by plate counts).

**The viability of*L. acidophilus*and*Bifidobacterium bifidum*in yoghurt samples**.

The changes in the viable counts of *L. acidophilus* and *B. bifidum* are presented in Table 1. Data explained that the counts of both *L. acidophilus* and *B. bifidum* cultures for all the yoghurt samples under study ranged between 8.76 and 9.75 log CFU/g and 8.94–9.75 log CFU/g respectively.

**Table 1:** Viable counts (Log CFU/g) of probiotic bacteria in different treatments during storage time

CSP (weight%)	0%			1%			3%			5%		
Day	1	11	21	1	11	21	1	11	21	1	11	21
PB	8.58.13 ± 0.01^cA^	8.90 ± 0.09^bA^	9.15 ± 0.08^bB^	9.13 ± 0.04^aC^	9.13 ± 0.03^aC^	9.05 ± 0.01^bA^	9.18 ± 0.06^aC^	9.03 ± 0.07 ^bB^	9.13 ± 0.01^bB^	8.94 ± 0.01^aB^	9.18 ± 0.03^aC^	9.75 ± 0.02^bC^
PL	8.76 ± 0.08^bA^	8.95 ± 0.01^bA^	9.33 ± 0.08^bA^	9.23 ± 0.01^bB^	9.39 ± 0.02^bB^	9.63 ± 0.01^cB^	9.33 ± 0.01^bC^	9.41 ± 0.08 ^cC^	9.70 ± 0.01^aC^	9.4 ± 0.06 ^bC^	9.50 ± 0.07^bC^	9.75 ± 0.04^bC^
PBL	8.34 ± 0.02^aA^	8.20 ± 0.08^aA^	7.39 ± 0.03^aA^	8.70 ± 0.31^aB^	9.05 ± 0.09^aC^	8.32 ± 0.23^aB^	9.09 ± 0.11^aC^	8.43 ± 0.08 ^aB^	9.15 ± 0.08^bC^	9.03 ± 0.06^aC^	9.15 ± 0.09^aC^	9.02 ± 0.01^aC^

Different uppercase letters in the same row and lowercase letters in the same column on the same days indicate significant differences between different treatments (Tukey’s test *p* < 0.05)

**CSP** (Coriander seed powder**)**; **PB**: yogurt sample with *Bifidobacterium bifidum*; **PL**: yogurt sample *Lactobacillus acidophilus*; **PBL**: yogurt sample with *Lactobacillus acidophilus* and *Bifidobacterium bifidum*

### Physicochemical properties

#### pH and acidity

There were significant (*P* < 0.05) differences in pH and acidity value of yogurt samples during storage time but all of them were in the acceptable range (Table 2). The treatments containing probiotic bacteria had a significantly lower pH level than the control sample (*p* < 0.05), but the addition of CSP in different concentrations did not cause a significant difference. The results of titrable acidity also showed that the treatment containing probiotic bacteria (PBL) has a significantly high level of acidity. (Table 2)

**Table 2:** Effect of different concentrations of coriander seed powder and probiotic bacteria in changes of pH and titratable acidity in yoghurt samples during the storage

Yogurt sample		CSP (weight%)			
**pH**	Storage period (days)	0	1	3	5
C	1	4.42 ± 0.23^cA^	4.28 ± 0.05^cA^	4.10 ± 0.22^bA^	3.99 ± 0.13^bA^
	11	4.24 ± 0.09^cA^	4.14 ± 0.02^cA^	4 ± 0.02^bA^	3.90 ± 0.23^bA^
	21	4.06 ± 0.18^cA^	4 ± 0.13^bA^	3.79 ± 0.14^aA^	3.73 ± 0.05^bA^
PB	1	3.93 ± 0.06^bA^	3.55 ± 0.09^aA^	3.76 ± 0.12^aA^	3.67 ± 0.19^aA^
	11	3.83 ± 0.13^bA^	3.45 ± 0.06^aA^	3.62 ± 0.14^aA^	3.53 ± 0.04^aA^
	21	3.42 ± 0.14^bA^	3.13 ± 0.13^aA^	3.52 ± 0.11^aA^	3.24 ± 0.07^bA^
PL	1	3.65 ± 0.07^aA^	4.13 ± 0.10^cC^	4.03 ± 0.07^bC^	3.98 ± 0.07^bB^
	11	3.51 ± 0.07^aA^	3.99 ± 0.11^abB^	3.89 ± 0.06^aB^	3.91 ± 0.11^bB^
	21	3.21 ± 0.08^aA^	3.86 ± 0.17^bA^	3.62 ± 0.06^aA^	3.54 ± 0.12^bA^
PBL	1	3.88 ± 0.11^bA^	3.76 ± 0.15^bA^	3.66 ± 0.07^aA^	3.64 ± 0.10^aA^
	11	3.83 ± 0.1^bA^	3.73 ± 0.03^aA^	3.63 ± 0.15^aA^	3.61 ± 0.06^aA^
	21	3.26 ± 0.06^bB^	3.48 ± 0.12^aA^	3.34 ± 0.17^aA^	3.26 ± 0.11^aA^
**TA**					
C	1	1.03 ± 0.03^aA^	1.2 ± 0.03^bB^	1.04 ± 0.03^bB^	1.03 ± 0.03^bB^
	11	1.05 ± 0.03^aA^	1.20 ± 0.03^aB^	1.14 ± 0.03^bB^	1.13 ± 0.03^bB^
	21	1.15 ± 0.03^aA^	1.35 ± 0.03^bB^	1.29 ± 0.03^bB^	1.38 ± 0.03^cB^
PB	1	1.2 ± 0.03^cB^	1.05 ± 0.03^cC^	1.07 ± 0.03^bB^	1.1 ± 0.03^bA^
	11	1.3 ± 0.03^cA^	1.25 ± 0.03^cB^	1.15 ± 0.05^bA^	1.18 ± 0.05^bA^
	21	1.35 ± 0.03^cB^	1.5 ± 0.03^cC^	1.37 ± 0.03^bA^	1.25 ± 0.10^bA^
PL	1	1.03 ± 0.03^bA^	1.11 ± 0.03^bB^	1.08 ± 0.03^bA^	1.07 ± 0.03^cA^
	11	1.11 ± 0.06^bA^	1.2 ± 0.05^aA^	1.17 ± 0.05^bA^	1.16 ± 0.05^bA^
	21	1.28 ± 0.03^bA^	1.36 ± 0.04^bB^	1.33 ± 0.04^bB^	1.32 ± 0.04^cB^
PBL	1	1.2 ± 0.03^dC^	1.05 ± 0.03^aB^	1.03 ± 0.03^aB^	1 ± 0.03^aA^
	11	1.28 ± 0.07^dB^	1.03 ± 0.07^bA^	1.01 ± 0.07^aA^	0.97 ± 0.07^aA^
	21	1.34 ± 0.05^dB^	1.19 ± 0.05^aA^	1.17 ± 0.05^aA^	1.13 ± 0.05^aA^

Different uppercase letters in the same row and lowercase letters in the same column on the same days indicate significant differences between different treatments (Tukey’s test *p* < 0.05)

**CSP (**Coriander seed powder**)**; **C**: control sample with starter culture; **PB**: yogurt sample with *Bifidobacterium bifidum*; **PL**: yogurt sample with *L. acidophilus*; **PBL**: yogurt sample with *Lactobacillus acidophilus* and *Bifidobacterium bifidum*

#### Water-holding capacity and syneresis

The water-holding capacity and syneresis of yogurt in all samples show significant difference (*p* < 0.05) and the highest water-holding capacity was determined in the yoghurt containing 1% CSP and both probiotic strains in this study on day 11 (Table 3).

**Table 3:** Synersis and water holding capacity of coriander seed powder yoghurt

Yogurt sample		CSP (weight%)			
**Synersis**	**Storage period (days)**	0	1	3	5
C	1	55.66 ± 1.52^aA^	53.02 ± 0.72^cC^	53.06 ± 0.88^aB^	51.12 ± 1.12^aA^
	11	61.77 ± 1.33^aB^	58.44 ± 1.22^aA^	57.1 ± 1.07^aA^	55.1 ± 0.56^aA^
	21	51.56 ± 1.04^aB^	48.23 ± 1.1^aA^	46.89 ± 0.49^aA^	44.89 ± 0.78^aA^
PB	1	61.66 ± 1.97^bB^	53.66 ± 1.55^bA^	53.66 ± 1.02^aA^	55.66 ± 1.33^bA^
	11	68.04 ± 1.19^aB^	61.44 ± 1.6^bA^	61.1 ± 0.97^bA^	61.77 ± 0.99^bA^
	21	58.53 ± 1.33^aB^	51.23 ± 1.12^bA^	50.99 ± 1.19^bA^	51.56 ± 0.78^bA^
PL	1	62 ± 2.02^cB^	57.33 ± 1.92^bA^	59.66 ± 1.32^bA^	58.33 ± 1.44^cA^
	11	67.19 ± 1.57^aC^	65.1 ± 1.66^bB^	62.1 ± 0.97^cA^	68.44 ± 0.85^cC^
	21	56.16 ± 1.16^aB^	53.89 ± 1.85^bA^	54.89 ± 1.25^cA^	58.23 ± 1.35^cB^
PBL	1	68.33 ± 1.5^dB^	64.2 ± 1.02^bA^	63 ± 2.02^cA^	65 ± 0.79^dA^
	11	75.1 ± 1.07^bB^	68.04 ± 1.17^bA^	70.44 ± 1.67^dA^	74.44 ± 0.63^dB^
	21	64.89 ± 0.5^bC^	60.23 ± 0.49^bB^	57.23 ± 1.15^dA^	64.23 ± 1.5^dC^
**WHC**					
C	1	38.11 ± 0.78^aA^	42.01 ± 1.08^aB^	44.77 ± 1.07^aC^	41.44 ± 0.83^aB^
	11	42.14 ± 2.08^aA^	49.29 ± 1.08^aC^	49.01 ± 1.32^aC^	47.67 ± 1.01^aB^
	21	36.34 ± 1.75^aA^	40.04 ± 1.05^aC^	40.01 ± 1.35^aC^	42.67 ± 1.06^aB^
PB	1	46.44 ± 0.74^bA^	46.44 ± 1.06^aA^	47.72 ± 0.69^bA^	47.11 ± 1.14^bA^
	11	50.67 ± 0.98^bA^	51.67 ± 0.58^aA^	52.01 ± 2.12^bA^	51.34 ± 2.0^bA^
	21	44.67 ± 1.06^bA^	44.67 ± 1.33^aA^	46.01 ± 1.25^bA^	45.34 ± 1.15^bA^
PL	1	42.44 ± 1.77^aA^	45.12 ± 1.51^aA^	45.12 ± 1.60^cA^	46.44 ± 1.22^bA^
	11	48.67 ± 2.08^aA^	49.34 ± 1.2^aA^	50.01 ± 1.14^bA^	50.67 ± 1.19^bA^
	21	42.67 ± 1.0 ^aA^	43.34 ± 1.75^aA^	44.01 ± 1.55^bA^	44.67 ± 1.14^bA^
PBL	1	37.11 ± 1.08^aA^	41.11 ± 1.6^aB^	45.19 ± 1.17^aC^	38.44 ± 1.77^aA^
	11	42.3 ± 0.73^aA^	46.03 ± 1.02^aB^	49.01 ± 1.15^aC^	42.67 ± 0.38^aA^
	21	40.34 ± 1.70^aB^	43.04 ± 1.55^aC^	43.05 ± 1.25^aC^	36.67 ± 1.06^aA^

Different uppercase letters in the same row and lowercase letters in the same column on the same days indicate significant differences between different treatments (Tukey’s test *p* < 0.05)

**CSP (**Coriander seed powder**)**; **C**: control sample with starter culture; **PB**: yogurt sample with *Bifidobacterium bifidum*; **PL**: yogurt sample with *L. acidophilus*; **PBL**: yogurt sample with *Lactobacillus acidophilus* and *Bifidobacterium bifidum.***WHC**: Water holding capacity

#### Yeast and mold

As data shows in Table 4. Colony counts between day 1 and day 21 are significantly different (*p* < 0.05). Our data declares coriander seed powder reduced yeast and mold colony counts during storage time, this may occur because of seed essential oils that proved to be a potential natural source of antifungal agent (Lasram et al. 2019).

**Table 4:** Yeast and mold count in yogurt samples

Yogurt sample	CSP (weight%)	Day		
		1	11	21
C	0	1.47 ± 0.28^bA^	1.87 ± 0.23^bA^	1.92 ± 0.22^aAB^
	1	1.55 ± 0.21^bA^	1.70 ± 0.18^cA^	1.82 ± 0.11^cA^
	3	1.26 ± 0.11^bA^	1.34 ± 0.17^bA^	1.46 ± 0.11^bA^
	5	1.21 ± 0.15^bA^	1.26 ± 0.10^bA^	1.39 ± 0.17^bA^
PB	0	1.12 ± 0.14^aA^	1.16 ± 0.27^aA^	1.37 ± 0.13^aAB^
	1	1.26 ± 0.15^aA^	1.26 ± 0.10^bA^	1.35 ± 0.24^cA^
	3	1.50 ± 0.22^cA^	1.52 ± 0.24^bA^	1.56 ± 0.1^cA^
	5	0.96 ± 0.10^bA^	1.06 ± 0.17^bA^	1.26 ± 0.09^bAB^
PL	0	1.28 ± 0.27^aA^	1.32 ± 0.11^aA^	1.38 ± 0.10^aA^
	1	0.92 ± 0.15^aA^	1.06 ± 0.0^bA^	1.22 ± 0.09^bAB^
	3	1.32 ± 0.10^bA^	1.38 ± 0.20^bA^	1.45 ± 0.10^bA^
	5	1.12 ± 0.08^bA^	1.17 ± 0.27^bA^	1.26 ± 0.13^bAB^
PBL	0	1.06 ± 0.22^aA^	1.26 ± 0.17^aA^	1.34 ± 0.07^aA^
	1	0.76 ± 0.24^aA^	0.96 ± 0^aA^	1.09 ± 0.11^aAB^
	3	0.88 ± 0.17^aA^	1.08 ± 0.13^aA^	1.06 ± 0.05^aAB^
	5	0. 32 ± 0.15^aA^	0.47 ± 0.08^aA^	0.64 ± 0.13^aAB^

Different uppercase letters in the same row and lowercase letters in the same column on the same days indicate significant differences between different treatments (Tukey’s test *p* < 0.05)

**CSP (**Coriander seed powder**)**; **C**: control sample with starter culture; **PB**: yogurt sample with *Bifidobacterium bifidum*; **PL**: yogurt sample with *L. acidophilus*; **PBL**: yogurt sample with *Lactobacillus acidophilus* and *Bifidobacterium bifidum*

#### Sensory properties

The average scores of all sensorial attributes of CSP yogurt samples are presented in Table 5. Among formulations scores that contain both CSP with *L. acidophilus* and *B. bifidum*, PBLG1, and PBLG3 yogurt samples (their values ranged between 0 and 50) significant changes (P < 0.05) have been observed.

**Table 5:** Sensory scores in yogurt samples

Yogurt sample	Mean score (overall acceptability)
C	38.50 ± 4.79^a^
CG1	43.43 ± 2.26^a^
CG3	41.86 ± 1.86^a^
CG5	42.13 ± 1.26^a^
PB	39.50 ± 4.66^a^
PL	41.00 ± 1.50^a^
PBG1	38.33 ± 3.74^a^
PBG3	39.86 ± 4.21^a^
PBG5	43.53 ± 2.41^a^
PBLG1	44.76 ± 2.73^a^
PBLG3	43.00 ± 2.47^a^
PBLG5	42.13 ± 2.37^a^
PBL	44.56 ± 2.48^a^
PLG1	42.93 ± 3.14^a^
PLG3	45.56 ± 2.07^a^
PLG5	41.96 ± 1.68^a^

* The mean values followed by the same letter in the column are non-significantly different (Tukey’s test p < 0.05)

Data compared with control sample (*P < 0.05*)

**CSP(**Coriander seed powder**)**; **C**: control sample with starter culture; **CG1**: yogurt sample containing 1% CSP with starter culture; **CG3**: yogurt sample containing 3% CSP with starter culture; **CG5** yogurt sample containing 5% CSP with starter culture; **PB**: yogurt sample with *Bifidobacterium bifidum*; **PL**: yogurt sample with *L. acidophilus*; **PBG1**: yogurt sample containing 1% CSP with *Bifidobacterium bifidum*; **PBG3**: yogurt sample containing 3% CSP with *Bifidobacterium bifidum*. **PBG5**: yogurt sample containing 5% CSP with *Bifidobacterium bifidum* ; **PBLG1**: yogurt sample containing 1% CSP with *Lactobacillus acidophilus* and *Bifidobacterium bifidum*; **PBLG3**: yogurt sample containing 3% CSP with *Lactobacillus acidophilus* and *Bifidobacterium bifidum*; **PBLG5**: yogurt sample containing 5% CSP with *Lactobacillus acidophilus* and *Bifidobacterium bifidum*; **PBL**: yogurt sample with *Lactobacillus acidophilus* and *Bifidobacterium bifidum* ; **PLG1**: yogurt sample containing 1% CSP with *L. acidophilus*; **PLG3**: yogurt sample containing 3% CSP with *Lactobacillus acidophilus*; **PLG5**: yogurt sample containing 5% CSP with *L. acidophilus*

## Discussion

The results of this study indicate that the addition of coriander seed powder (CSP) and probiotic strains to yoghurt can improve its nutritional and functional properties. The chemical composition analysis of CSP showed that it is a good source of protein, fiber, and ash. The growth of probiotic bacteria in MRS broth and agar was confirmed by visible growth, and their viability in yoghurt samples was maintained at high levels during the storage period.

The viability of probiotic cultures has been influenced by different factors such as the strains used, the interaction between species present, culture conditions, production of hydrogen peroxide due to bacterial metabolism, final acidity of the product and the concentrations of lactic and acetic acids in yogurt and fermented milk products. Furthermore, the interaction between microbial cultures in the product can affect the growth of the yogurt starter cultures and probiotic viability (Nyanzi et al. 2021). Probiotics are one of the most important functional food substances and admitted beneficial health effects on the host when consumed in adequate amounts (defined as live microorganisms) (Yilmaz-Ersan et al. 2020). The most important factors in fermented dairy products containing probiotics are the viability of the probiotics and the sensory and physical properties changes that may occur (Mani-López et al. 2014).

In the current study viability of two probiotic bacteria, *L. acidophilus* and *B. bifidum* was assessed.

Available shreds of evidence indicate that there is a positive correlation between the presence of coriander seed powder and viable counts of probiotic bacteria in yoghurt samples during storage. The changes in the viable counts of *L. acidophilus* and *B. bifidum* are presented in Table 1.

Data explained that the counts of both *L. acidophilus* and *B. bifidum* cultures for all the yoghurt samples under study ranged between 8.76 and 9.75 log CFU/g and 8.94–9.75 log CFU/g respectively. There was a slight decrease in the culture counts of PB (yogurt sample with *Bifidobacterium bifidum*) in comparison with PBG1 yogurt (yogurt sample containing 1% coriander seed powder with *Bifidobacterium bifidum*), but the culture counts of PBG3 (yogurt sample containing 3% coriander seed powder with *Bifidobacterium bifidum*) and PBG5 yogurt samples were higher in comparison with PBG1 yogurt sample During the first day. During storage, the counts of *L. acidophilus* in PL yogurt samples remained almost stable. PBLG1 yogurt (yogurt sample containing 1% CSP with *L. acidophilus* and *Bifidobacterium bifidum*) 11 days after inoculation was higher in probiotic counts and decreased on day 21. Adding 3% and 5% of coriander seed powder to probiotic yogurt increased the bacterial count of the samples. As data shows all the yogurt sample formulae were in the acceptable range of live probiotic bacteria, therefore coriander seed powder helps probiotic yogurt to survive *L. acidophilus* and *Bifidobacterium bifidum* and keep them alive. These results are consistent with the findings of shariati (2020), who found increase in the viability of probiotics in the presence of 0.05% of coriander extract. The coriander leaf extracts due to prebiotic compounds including phenolic compounds and remarkable values of carbohydrates and water-soluble vitamins can improve the viability of probiotic bacteria during cold storage. (Shahwar et al. 2012). In another study Haji Ghafarloo et al. (2019) stated that ginger extract at low concentration acts as prebiotic to promote the *Bifidobacterium bifidum* growth. According to Abdel-Salam et al. results, total polyphenols in coriander extract (23.85 mg gallic acid dL^− 1^) had more antioxidant capacity than probiotic supernatant alone. The uptake of probiotic fermented milk fortified with a rich source or combined source of natural fibers such as coriander seeds (probiotic-herbal mixture), represents promising approaches to body health (Abdel-Salam et al. 2018). Polyphenols are secondary metabolites of plants, ranging from that of a simple phenolic molecule to that of a complex high-molecular mass polymer and show strong evidence to have a prebiotic effect on preclinical studies (Abdel-Salam et al. 2018).

The pH of samples significantly decreased during the storage time, while the acidity increased (*p* < 0.05). Shariati et al. (2020) reported a similar trend for Doogh samples was investigated. According to several researchers, this increase could be due to the production of lactic acid and other organic acids by the lactic acid culture. (Barrros et al., 2019; Chen et al. 2017).

In this study, the decrease in pH and increase in acidity was more in samples containing CSP which is consistent with the results of Shariati et al. in Doogh formulated with *Lactobacillus plantarum* LS5, cress seed gum, and coriander leaves extract (Shariati et al. 2020). The reason can be the better growth of probiotic bacteria and the production of lactic acid. The viability and metabolic features of probiotic bacteria are strongly influenced by pH and titratable acidity. According to Tripathi and Giri (2014), the ideal pH for optimum growth of bifidobacteria and lactobacillus species varies between 6.00 and 7.00 and 5.50–6.09, respectively. Starter cultures in yogurt produce acid, which is the major reason for the decline of probiotic viability during storage. The tolerance of strains to acid is related to their survival in yogurt with a pH range of 3.7 to 4.3. Lactobacilli are more acid tolerant due to possessing a pH homeostasis system (Meybodi et al. 2020).

Syneresis index (serum release) along with WHC in yogurt is unfavorable and is considered as an indicator of yogurt quality during storage. The water holding capacity and synergistic results showed that adding coriander powder up to a concentration of 3% significantly (*p* < 0.05) had a positive effect on the samples compared to the control sample and treatment with a concentration of 5%. Furthermore, initial syneresis of yogurts decreased with time, which was in line with the findings of Korkmaz et al. (2012).

Probiotic cells can also be incorporated in yogurt via immobilization in natural supports including fruits and grains. For instance, in one study, yogurt supplemented with immobilized *L. casei* on fresh apple pieces, wheat grains or dried raisins presented less syneresis (appearance of liquid on the milk gel surfaces and gel shrinkage) due to their water holding capacity. (Bosnea et al. 2017).

Although milk is pasteurized before yogurt production, contamination with yeasts in yogurt occurs during production processes and can be problematic in yogurts containing herbal additives. (Trigueros et al. 2012). Numbers of yeasts and molds were significantly higher in control yogurts. Counts of yeasts and molds increased (*p* < 0.05) in yogurts during cold storage (Table 4) and the highest level was 1.92 cfu/g at day 21 in control yogurts. The colony counts of yeast and mold were significantly reduced in CSP yoghurt samples, possibly due to the antifungal properties of coriander seed essential oils. The effect of antimicrobial compounds in yogurt has also been observed in similar studies by Shahbazi and Shavisi (2019) using oregano methanolic extract. However, in another study, due to the contamination of the ingredients (date) added to yogurt, the count of mold and yeast was higher than the control sample (Trigueros et al. 2012).

In order to obtain useful information about the future commercial potential of a newly developed food, it is essential to conduct consumer sensory tests, such as the one performed in this study. It is possible that the addition of various plant and fruit compounds to a dairy product has a negative effect on the sensory characteristics. In the present research, sensory evaluation showed that yogurt samples containing CSP and probiotic strains did not have a negative effect on the sensory characteristics of the produced yogurts. These findings suggest that CSP and probiotics can be used as functional ingredients to enhance the nutritional value and sensory quality of yoghurt.

Furthermore, studies have shown that the use of probiotics and prebiotics together can improve symptoms of irritable bowel syndrome, reduce the risk of antibiotic-associated diarrhea, and even improve mental health outcomes such as anxiety and depression. Therefore, incorporating both probiotics and prebiotics into one’s diet can have a positive impact on overall health and well-being. These can be taken into consideration in further research on the use of synbiotic food products (Mahajan and Manjot, 2022).

Our results demonstrated that the addition of coriander seed powder (1,3 and 5% w/w) in probiotic yogurt containing *Lactobacillus acidophilus* and *Bifidobacterium bifidum* culture can improve the viability of *L. acidophilus* and *B. bifidum* during 21 days of storage) while physicochemical properties are in acceptable range.

It can be concluded that a mixture of probiotic strains (*L. acidophilus* and *B. bifidum*) with coriander seed powder in an optimal concentration can be introduced as a new synbiotic yogurt with suitable characteristics.

## Data Availability

All data generated or analyzed during this study are included in this manuscript.

## List of Abbreviations

CSP: Coriander seed powder
*L. acidophilus*: *Lactobacillus acidophilus*
*B. bifidum*: *Bifidobacterium bifidum*
PB: Yogurt sample with *Bifidobacterium bifidum*
PL: Yogurt sample with *L. acidophilus*
PBL: Yogurt sample with *Lactobacillus acidophilus* and *Bifidobacterium bifidum*
WHC: Water holding capacity
PBG1: Yogurt sample containing 1% CSP with *Bifidobacterium bifidum*
PBG3: Yogurt sample containing 3% CSP with *Bifidobacterium bifidum*
PBG5: Yogurt sample containing 5% CSP with *Bifidobacterium bifidum*
PBLG1: Yogurt sample containing 1% CSP with *Lactobacillus acidophilus* and *Bifidobacterium bifidum*
PBLG3: Yogurt sample containing 3% CSP with *Lactobacillus acidophilus* and *Bifidobacterium bifidum*
PBLG5: Yogurt sample containing 5% CSP with *Lactobacillus acidophilus* and *Bifidobacterium bifidum*
PLG1: Yogurt sample containing 1% CSP with *L. acidophilus*
PLG3: Yogurt sample containing 3% CSP with *Lactobacillus acidophilus*
PLG5: Yogurt sample containing 5% CSP with *L. acidophilus*
